# Association Between Raised HbA1c Levels and Hemorrhagic Transformation in Patients With Ischemic Stroke

**DOI:** 10.7759/cureus.19935

**Published:** 2021-11-27

**Authors:** Benish Afzal, Syed Ahsan Ali, Bushra Jamil

**Affiliations:** 1 Department of Internal Medicine, Aga Khan University Hospital, Karachi, PAK

**Keywords:** cerebro-vascular accident (stroke), hba1c, hemorrhagic transformation, ischemic stroke, diabetes mellitus type 2

## Abstract

Background

Glycated hemoglobin (HbA1c) is a commonly used indicator of glycemic control in diabetes mellitus. Uncontrolled diabetes can lead to cardiovascular complications. Ischemic strokes are often complicated by hemorrhagic transformation, which is the conversion of an infarcted area into an area of hemorrhage. The purpose of this study is to determine the association of raised HbA1c levels with the occurrence of hemorrhagic transformation in ischemic stroke.

Methods

This is a prospective, single-center cohort study of patients admitted to the Neurology and Medicine wards between June 1 and November 30, 2019. Inclusion criteria included adult patients who were admitted with acute ischemic stroke and had been tested for HbA1c on presentation. There were 110 ischemic stroke patients enrolled in our database. An HbA1c level >53 mmol/mol was considered raised. A comparison was done between the group with raised HbA1c levels and the group with target levels of HbA1c for the occurrence of hemorrhagic transformation. Brain imaging was used to diagnose hemorrhagic transformation.

Results

Out of 110 study participants with ischemic stroke, 70 (63.6%) patients had a history of prior known diabetes. The mean HbA1c levels were 7.44 ± 1.79%. A total of 77% of diabetic patients had raised HbA1c levels (>7%). Hemorrhagic transformation was seen in 21 (19.1%) patients, of whom only 38% (n=8) had raised HbA1c levels. The association between raised HbA1c and hemorrhagic transformation was not found to be statistically significant (p = 0.225).

Conclusion

In this study of patients with ischemic stroke, raised HbA1c levels were not found to be associated with hemorrhagic transformation. There is a need for larger scale studies to find out the cause and mechanism of hemorrhagic transformation in ischemic stroke.

## Introduction

Diabetes mellitus is a chronic non-communicable disease with a rising global prevalence, causing widespread disability and death [1]. International Diabetes Federation estimated the global diabetes prevalence to be 10.2% (578 million) by 2030 and 10.9% (700 million) by 2045 [1]. Cardiovascular diseases are the most common cause of morbidity and mortality in diabetic patients [2,3]. Poor nutrition, obesity, inadequate physical activity, urbanization, along with a rise in the proportion of older individuals in the global population are few of the factors leading to a rise in the incidence of diabetes and diabetes-related complications.

There is an increased risk of stroke in patients with diabetes [2]. Emerging Risk Factors Collaboration showed that the adjusted hazard ratios with diabetes were 2.27 (1.95-2.65) for ischemic stroke [4]. Poor glycemic control prior to ischemic stroke is an independent risk factor for higher mortality, increased stroke severity and unfavorable long-term functional outcomes [5]. Acute ischemic stroke is sometimes complicated by hemorrhagic transformation, which is bleeding into an area of ischemic brain after a stroke that can cause increased morbidity and mortality. Hemorrhagic transformation occurs in as many as 10% to 15% of patients with ischemic stroke [6]. Parenchymal hematoma involving more than 30% of the infarcted area is associated with clinical deterioration after 24 hours of stroke and increased mortality at three months [7]. Furthermore, the presence of hemorrhagic transformation interferes with treatments such as thrombolysis, anticoagulation and antiplatelet medications.

In Pakistan, there are a limited number of studies regarding the prevalence of diabetes, but a systemic analytic study shows the prevalence of 11.77% [8]. There is also a high burden of stroke that is increasing [9]. An increase in trends of ischemic strokes and diabetes has led to glycated hemoglobin (HbA1c) becoming an important topic of recent studies. A raised HbA1c level is associated with poor outcomes in stroke, causing higher six-month mortality and length of stay [10,11]. The purpose of our study is to determine the association of raised HbA1c levels with the occurrence of hemorrhagic transformation in ischemic stroke, so that physicians can be more vigilant in maintaining glycemic control and detecting hemorrhagic conversion in patients with uncontrolled diabetes. Furthermore, physicians will be more cautious in using dual antiplatelet or anticoagulants in such patients, if required.

## Materials and methods

This study was a prospective observational cohort study, conducted at the Internal Medicine department of a tertiary care hospital in Pakistan, Aga Khan University Hospital, Karachi. Patients from Neurology and Medicine wards were recruited from June to November 2019. This study was approved as an exemption by the Ethics Review Committee of Aga Khan University Hospital (approval number 2019-2008-5385). It was conducted according to the ethical guidelines of the Declaration of Helsinki. Patients’ strict confidentiality was maintained and information was anonymized and deidentified before the analysis. The informed consent of the patients was waived.

Patients of age more than 18 years, who were admitted with acute ischemic stroke and tested for HbA1c on presentation, were included in this study. Ischemic stroke was defined as the sudden loss of blood circulation to an area of the brain, resulting in a corresponding loss of neurologic function, diagnosed on CT scans or MRI imaging of brain. Those patients who were on anticoagulation at the time of presentation were excluded. There were 110 ischemic stroke patients enrolled in our database after we excluded 19 patients who were on anticoagulation prior to admission due to atrial fibrillation. HbA1c levels were checked on admission, and a level >53 mmol/mol (7%) was considered raised.

Demographics and clinical details, including age, gender, prior history of diabetes and hypertension, anticoagulation in-home medication, thrombolytic agent if given during hospital stay, length of stay and inpatient mortality, were collected. We recorded findings of subsequent CT scans and MRI scans of patients during the same hospital stay. Hemorrhagic transformation was described as ‘any degree of hyperdensity within the area of low attenuation’ in a CT scan. In MRI scans, hemorrhagic conversion was defined as a signal dropout in the susceptibility weighted imaging (SWI) sequence, in an area of acute ischemic stroke.

The sample size included 90 ischemic stroke patients and was calculated taking into account 95% confidence interval, 80% power, and reported prevalence of raised HbA1c to be 35% and normal HbA1c 65% in patients with ischemic stroke [12]. Patients enrolled in this study were divided into two groups according to the presence or absence of raised HbA1c levels. The occurrence of hemorrhagic transformation as an outcome was compared between the two groups. Statistical analysis was done with quantitative variables reported as means and standard deviations, and categorical variables reported as absolute numbers and percentages. Data was stratified by age, gender, diabetes, hypertension, HbA1c level and use of thrombolytic agent. A post-stratification chi-square test was applied. A p-value <0.05 was considered as statistically significant. To detect significant differences between groups, the chi-square test or the Fisher’s exact test was used for categorical variables.

## Results

In this study, out of 110 participants with ischemic stroke, 68.2% (n=75) were male. The mean age of the patients was 62.1 ± 13.8 years. Patients suffering from diabetes and hypertension were 70 (63.6%) and 85 (77.3%), respectively. The mean HbA1c levels were 7.44 ± 1.79%; 77% of diabetic patients had raised HbA1c levels (>7%).

**Figure 1:**
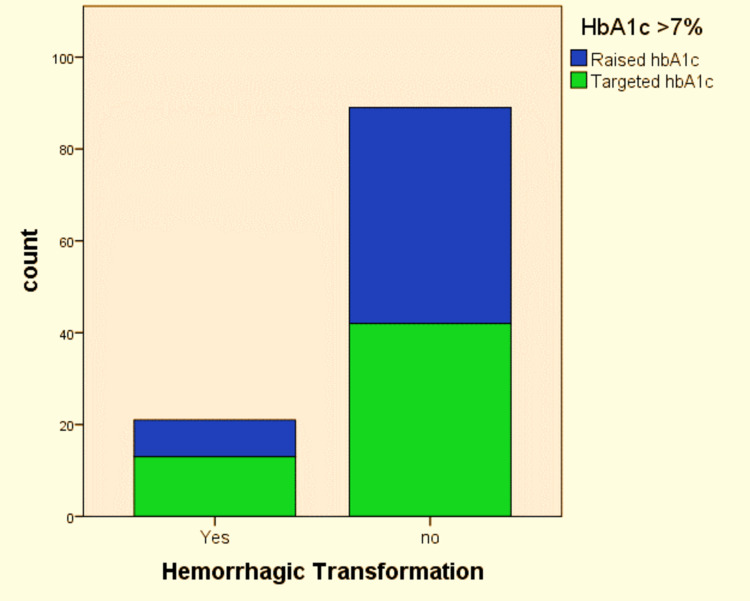
Association of raised HbA1c levels with hemorrhagic transformation in patients admitted with ischemic stroke

The association between raised HbA1c levels and hemorrhagic transformation was not found to be statistically significant (p = 0.225). Hemorrhagic transformation was seen in 21 (19.1%) patients, of whom only 38% (n=8) had raised HbA1c levels, as shown in Table 1 and Figure 1.

**Table 1:** Analysis of risk factors of HT after ischemic stroke HT = hemorrhagic transformation; CI = confidence interval; TPA = tissue plasminogen activator

Variables		Number	HT, n (%)	Odds ratio	95% CI	p value
Age ≥ 45 years	Yes	100	19 (19%)	0.938	0.18-4.78	
	No	10	2 (2%)			
Gender	Male	75	15 (20%)	1.208	0.43-3.44	0.722
	Female	35	6 (17%)			
Diabetes	Yes	70	12 (17.1%)	0.713	0.27-1.88	0.492
	No	40	9 (22.5%)			
Raised HbA1c on admission	Yes	55	8 (14.5%)	0.55	0.21-1.46	0.225
	No	55	13 (23.6%)			
Hypertension	Yes	85	18 (22.1%)	1.97	0.53-7.33	
	No	25	3 (12%)			
TPA given during hospital stay	Yes	5	0 (0%)			
	No	105	21 (20%)			

The odds ratio of developing hemorrhagic transformation with raised HbA1c was found to be 0.55 (95% confidence interval 0.21-1.46) and relative risk was 0.615. The mean length of hospital stay was 4.38 ± 3.79 days while inpatient mortality was 6.4% (n=7). In 71.4% of cases with inpatient mortality, HbA1c was found to be raised. Multivariate cox regression analysis is shown in Table 2. The hazard ratio of hemorrhagic transformation was 0.739 (p value was not found to be significant).

**Table 2:** Multivariate cox regression analysis CI = confidence interval

		Inpatient mortality		Hazard ratio	95% CI for hazard ratio
		Yes, n (%)	No, n (%)		
Hemorrhagic transformation	Yes	2 (9.5%)	19 (90.5%)	0.739	0.13-4.25
	No	5 (5.6%)	84 (94.4%)		
Raised HbA1c	Yes	5 (9.1%)	50 (90.9%)	0.984	0.68-1.42
	No	2 (3.6%)	53 (96.4%)		
Age ≥ 45 years	Yes	6 (6%)	94 (94%)	1.04	0.98-1.10
	No	1 (10%)	9 (90%)		

## Discussion

In this prospective, observational cohort study, we found that there is no association of raised HbA1c levels with the occurrence of hemorrhagic transformation in patients with ischemic stroke. Larger scale studies are needed to confirm this finding. However, this study established that most diabetic patients presenting with ischemic stroke had failed to achieve targeted HbA1c levels.

There are a limited number of South Asian studies exploring causes and mechanism of hemorrhagic transformation. In contrast to our study, some studies done in China suggested an association between raised HbA1c and hemorrhagic transformation [13,14]. In a prospective cohort study conducted in Lianyungang Hospital in China, 426 patients with anterior ischemic stroke were included and HbA1c was found to be a predictor of hemorrhagic transformation [13]. Another study conducted on 287 patients from the First Affiliated Hospital of Wenzhou Medical University showed that a high stress hyperglycemia ratio (fasting blood sugar divided by HbA1c) was significantly associated with an increased risk of hemorrhagic transformation in patients with ischemic stroke [15].

In a retrospective study conducted by Sung et al., which was done in hospitals affiliated with Taipei Medical University, an association between raised HbA1c levels and a poor clinical outcome after ischemic stroke was determined. In this study, 484 patients with acute ischemic stroke were included and it was found that HbA1c is not a significant predictor of a poor neurological outcome, although random and fasting glucose were found to be statistically significant predictors [16].

Risk factors for hemorrhagic transformation include a history of use of tissue plasminogen activator, raised blood pressures and size of infarct [17]. The exact pathophysiological mechanism through which hyperglycemia may cause hemorrhagic transformation in stroke patients is unknown. Many factors influence blood glucose levels, so there is a variability in glucose levels throughout the day. In addition, the time interval from the onset of stroke symptoms to the measurement of blood glucose might vary from one patient to another. HbA1c is relatively more stable than acute blood glucose and widely used for monitoring.

The limitations of our study were that different modalities of brain imaging were used as some cases had MRI done while other cases had CT scans. The infarct size was not taken into account. The cases were admitted on variable days from the date of symptom onset and there was loss to follow-up after discharge; many of them did not have repeat imaging done after discharge.

## Conclusions

In this study, raised HbA1c was not found to be associated with hemorrhagic transformation in patients with ischemic stroke in our population. There is a need for larger scale studies to find out the cause and mechanism of hemorrhagic transformation in ischemic stroke. This will enable physicians to find ways to prevent such complications and will guide its management, reducing morbidity and disability associated with it.
